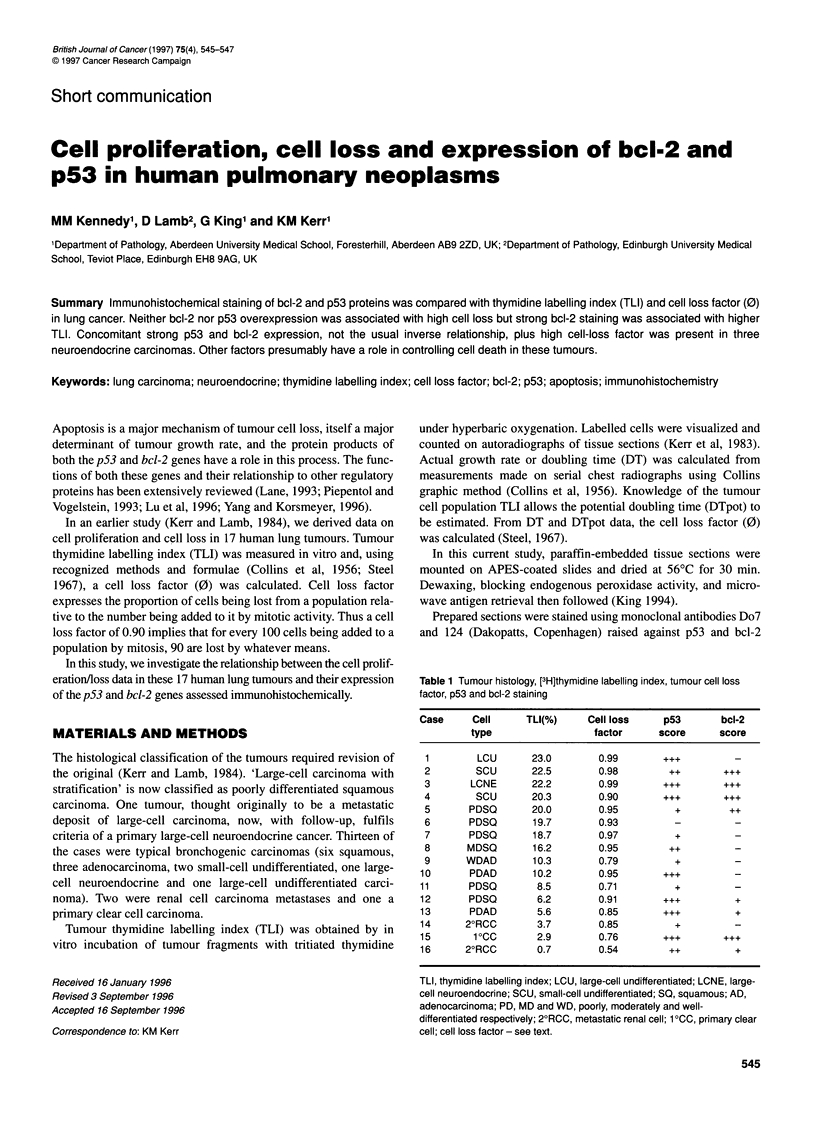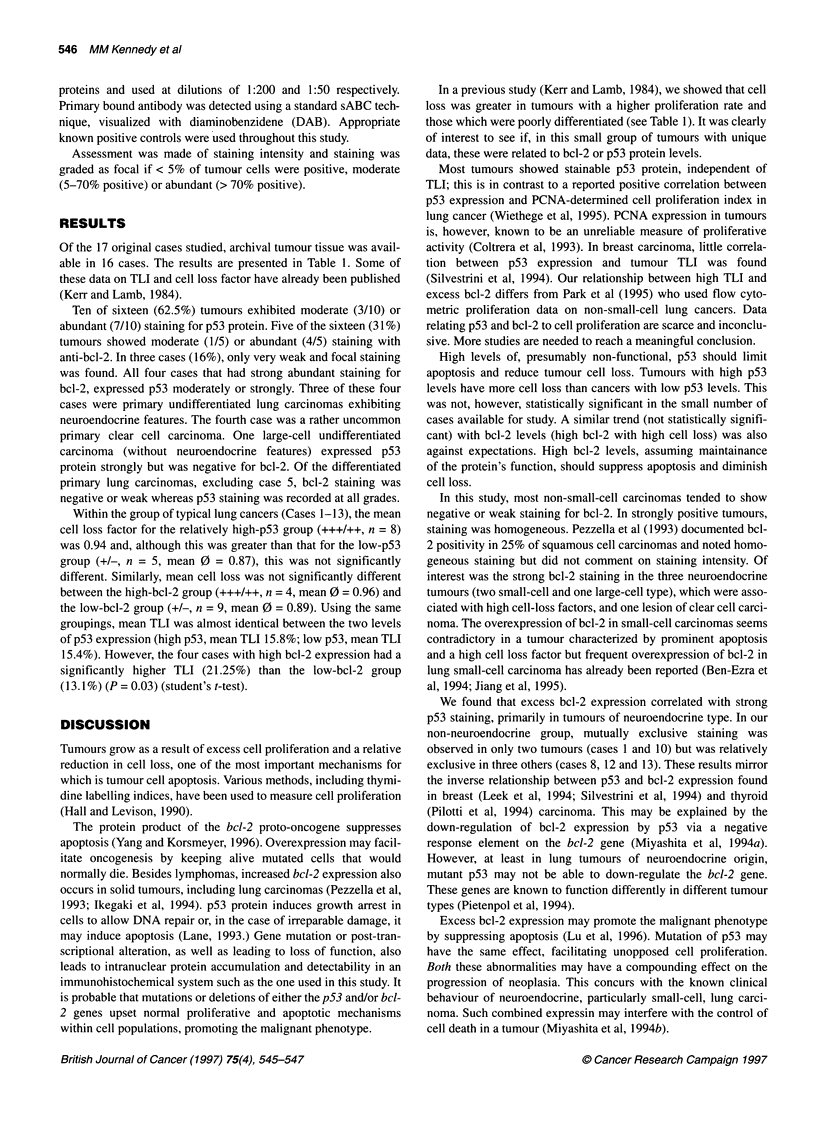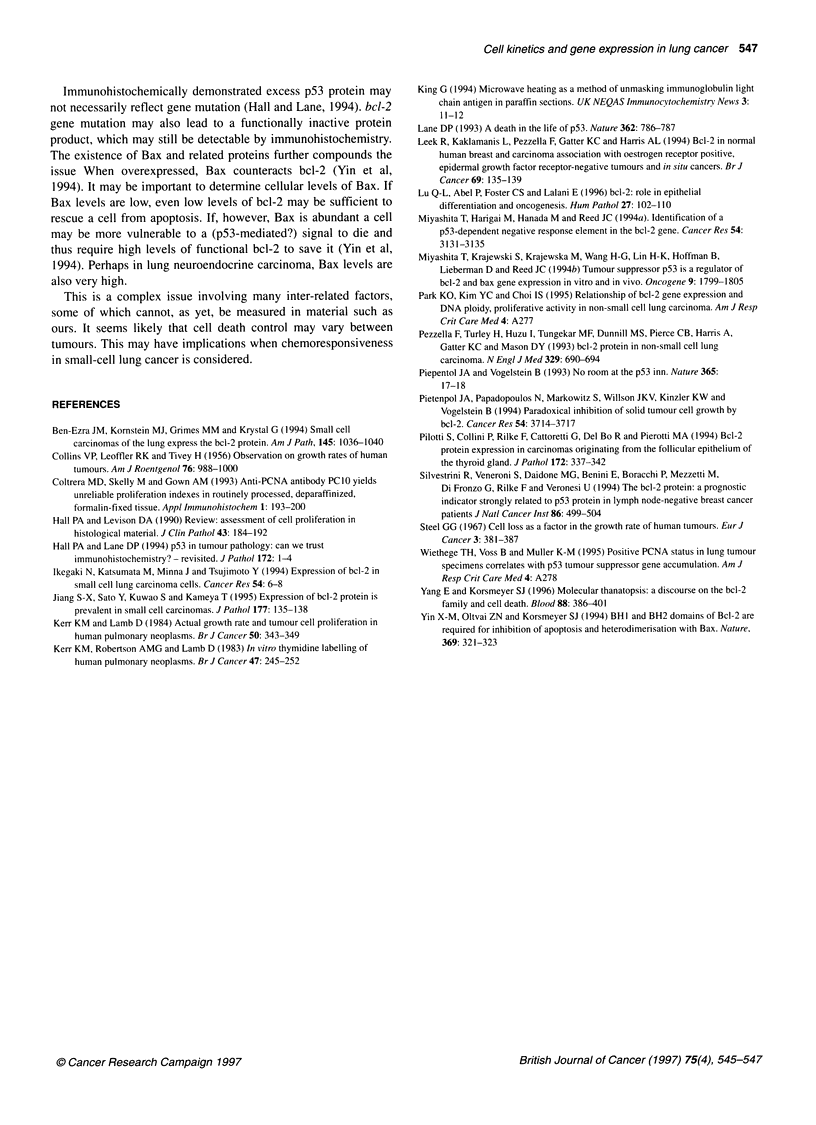# Cell proliferation, cell loss and expression of bcl-2 and p53 in human pulmonary neoplasms.

**DOI:** 10.1038/bjc.1997.95

**Published:** 1997

**Authors:** M. M. Kennedy, D. Lamb, G. King, K. M. Kerr

**Affiliations:** Department of Pathology, Aberdeen University Medical School, Foresterhill, UK.

## Abstract

Immunohistochemical staining of bcl-2 and p53 proteins was compared with thymidine labelling index (TLI) and cell loss factor (O) in lung cancer. Neither bcl-2 nor p53 overexpression was associated with high cell loss but strong bcl-2 staining was associated with higher TLI. Concomitant strong p53 and bcl-2 expression, not the usual inverse relationship, plus high cell-loss factor was present in three neuroendocrine carcinomas. Other factors presumably have a role in controlling cell death in these tumours.


British Journal of Cancer (1997) 75(4), 545–547
© 1997 Cancer Research Campaign

Short communication

### Cell proliferation, cell loss and expression of bcl-2 and p53 in human pulmonary neoplasms

**MM Kennedy[1], D Lamb[2], G King[1] and KM Kerr[1]**

[1]Department of Pathology, Aberdeen University Medical School, Foresterhill, Aberdeen AB9 2ZD, UK; [2]Department of Pathology, Edinburgh University Medical School, Teviot Place, Edinburgh EH8 9AG, UK

**Summary** Immunohistochemical staining of bcl-2 and p53 proteins was compared with thymidine labelling index (TLI) and cell loss factor (Ø) in lung cancer. Neither bcl-2 nor p53 overexpression was associated with high cell loss but strong bcl-2 staining was associated with higher TLI. Concomitant strong p53 and bcl-2 expression, not the usual inverse relationship, plus high cell-loss factor was present in three neuroendocrine carcinomas. Other factors presumably have a role in controlling cell death in these tumours.

**Keywords:** lung carcinoma; neuroendocrine; thymidine labelling index; cell loss factor; bcl-2; p53; apoptosis; immunohistochemistry

Apoptosis is a major mechanism of tumour cell loss, itself a major determinant of tumour growth rate, and the protein products of both the *p53* and *bcl-2* genes have a role in this process. The functions of both these genes and their relationship to other regulatory proteins has been extensively reviewed (Lane, 1993; Piepentol and Vogelstein, 1993; Lu et al, 1996; Yang and Korsmeyer, 1996).

In an earlier study (Kerr and Lamb, 1984), we derived data on cell proliferation and cell loss in 17 human lung tumours. Tumour thymidine labelling index (TLI) was measured in vitro and, using recognized methods and formulae (Collins et al, 1956; Steel 1967), a cell loss factor (Ø) was calculated. Cell loss factor expresses the proportion of cells being lost from a population relative to the number being added to it by mitotic activity. Thus a cell loss factor of 0.90 implies that for every 100 cells being added to a population by mitosis, 90 are lost by whatever means.

In this study, we investigate the relationship between the cell proliferation/loss data in these 17 human lung tumours and their expression of the *p53* and *bcl-2* genes assessed immunohistochemically.

### MATERIALS AND METHODS

The histological classification of the tumours required revision of the original (Kerr and Lamb, 1984). 'Large-cell carcinoma with stratification' is now classified as poorly differentiated squamous carcinoma. One tumour, thought originally to be a metastatic deposit of large-cell carcinoma, now, with follow-up, fulfils criteria of a primary large-cell neuroendocrine cancer. Thirteen of the cases were typical bronchogenic carcinomas (six squamous, three adenocarcinoma, two small-cell undifferentiated, one large-cell neuroendocrine and one large-cell undifferentiated carcinoma). Two were renal cell carcinoma metastases and one a primary clear cell carcinoma.

Tumour thymidine labelling index (TLI) was obtained by in vitro incubation of tumour fragments with tritiated thymidine under hyperbaric oxygenation. Labelled cells were visualized and counted on autoradiographs of tissue sections (Kerr et al, 1983). Actual growth rate or doubling time (DT) was calculated from measurements made on serial chest radiographs using Collins graphic method (Collins et al, 1956). Knowledge of the tumour cell population TLI allows the potential doubling time (DTpot) to be estimated. From DT and DTpot data, the cell loss factor (Ø) was calculated (Steel, 1967).

In this current study, paraffin-embedded tissue sections were mounted on APES-coated slides and dried at 56°C for 30 min. Dewaxing, blocking endogenous peroxidase activity, and microwave antigen retrieval then followed (King 1994).

Prepared sections were stained using monoclonal antibodies Do7 and 124 (Dakopatts, Copenhagen) raised against p53 and bcl-2

**Table 1** Tumour histology, [$^3$H]thymidine labelling index, tumour cell loss factor, p53 and bcl-2 staining

| Case | Cell type | TLI(%) | Cell loss factor | p53 score | bcl-2 score |
|---|---|---|---|---|---|
| 1 | LCU | 23.0 | 0.99 | +++ | – |
| 2 | SCU | 22.5 | 0.98 | ++ | +++ |
| 3 | LCNE | 22.2 | 0.99 | +++ | +++ |
| 4 | SCU | 20.3 | 0.90 | +++ | +++ |
| 5 | PDSQ | 20.0 | 0.95 | + | ++ |
| 6 | PDSQ | 19.7 | 0.93 | – | – |
| 7 | PDSQ | 18.7 | 0.97 | + | – |
| 8 | MDSQ | 16.2 | 0.95 | ++ | – |
| 9 | WDAD | 10.3 | 0.79 | + | – |
| 10 | PDAD | 10.2 | 0.95 | +++ | – |
| 11 | PDSQ | 8.5 | 0.71 | + | – |
| 12 | PDSQ | 6.2 | 0.91 | +++ | + |
| 13 | PDAD | 5.6 | 0.85 | +++ | + |
| 14 | 2°RCC | 3.7 | 0.85 | + | – |
| 15 | 1°CC | 2.9 | 0.76 | +++ | +++ |
| 16 | 2°RCC | 0.7 | 0.54 | ++ | + |

TLI, thymidine labelling index; LCU, large-cell undifferentiated; LCNE, large-cell neuroendocrine; SCU, small-cell undifferentiated; SQ, squamous; AD, adenocarcinoma; PD, MD and WD, poorly, moderately and well-differentiated respectively; 2°RCC, metastatic renal cell; 1°CC, primary clear cell; cell loss factor – see text.

*Received 16 January 1996*
*Revised 3 September 1996*
*Accepted 16 September 1996*

*Correspondence to*: KM Kerr proteins and used at dilutions of 1:200 and 1:50 respectively. Primary bound antibody was detected using a standard sABC technique, visualized with diaminobenzidene (DAB). Appropriate known positive controls were used throughout this study.

Assessment was made of staining intensity and staining was graded as focal if < 5% of tumour cells were positive, moderate (5–70% positive) or abundant (> 70% positive).

### RESULTS

Of the 17 original cases studied, archival tumour tissue was available in 16 cases. The results are presented in Table 1. Some of these data on TLI and cell loss factor have already been published (Kerr and Lamb, 1984).

Ten of sixteen (62.5%) tumours exhibited moderate (3/10) or abundant (7/10) staining for p53 protein. Five of the sixteen (31%) tumours showed moderate (1/5) or abundant (4/5) staining with anti-bcl-2. In three cases (16%), only very weak and focal staining was found. All four cases that had strong abundant staining for bcl-2, expressed p53 moderately or strongly. Three of these four cases were primary undifferentiated lung carcinomas exhibiting neuroendocrine features. The fourth case was a rather uncommon primary clear cell carcinoma. One large-cell undifferentiated carcinoma (without neuroendocrine features) expressed p53 protein strongly but was negative for bcl-2. Of the differentiated primary lung carcinomas, excluding case 5, bcl-2 staining was negative or weak whereas p53 staining was recorded at all grades.

Within the group of typical lung cancers (Cases 1–13), the mean cell loss factor for the relatively high-p53 group (+++/++, $n$ = 8) was 0.94 and, although this was greater than that for the low-p53 group (+/–, $n$ = 5, mean Ø = 0.87), this was not significantly different. Similarly, mean cell loss was not significantly different between the high-bcl-2 group (+++/++, $n$ = 4, mean Ø = 0.96) and the low-bcl-2 group (+/–, $n$ = 9, mean Ø = 0.89). Using the same groupings, mean TLI was almost identical between the two levels of p53 expression (high p53, mean TLI 15.8%; low p53, mean TLI 15.4%). However, the four cases with high bcl-2 expression had a significantly higher TLI (21.25%) than the low-bcl-2 group (13.1%) ($P$ = 0.03) (student's $t$-test).

### DISCUSSION

Tumours grow as a result of excess cell proliferation and a relative reduction in cell loss, one of the most important mechanisms for which is tumour cell apoptosis. Various methods, including thymidine labelling indices, have been used to measure cell proliferation (Hall and Levison, 1990).

The protein product of the *bcl-2* proto-oncogene suppresses apoptosis (Yang and Korsmeyer, 1996). Overexpression may facilitate oncogenesis by keeping alive mutated cells that would normally die. Besides lymphomas, increased *bcl-2* expression also occurs in solid tumours, including lung carcinomas (Pezzella et al, 1993; Ikegaki et al, 1994). p53 protein induces growth arrest in cells to allow DNA repair or, in the case of irreparable damage, it may induce apoptosis (Lane, 1993.) Gene mutation or post-transcriptional alteration, as well as leading to loss of function, also leads to intranuclear protein accumulation and detectability in an immunohistochemical system such as the one used in this study. It is probable that mutations or deletions of either the *p53* and/or *bcl-2* genes upset normal proliferative and apoptotic mechanisms within cell populations, promoting the malignant phenotype.

In a previous study (Kerr and Lamb, 1984), we showed that cell loss was greater in tumours with a higher proliferation rate and those which were poorly differentiated (see Table 1). It was clearly of interest to see if, in this small group of tumours with unique data, these were related to bcl-2 or p53 protein levels.

Most tumours showed stainable p53 protein, independent of TLI; this is in contrast to a reported positive correlation between p53 expression and PCNA-determined cell proliferation index in lung cancer (Wiethege et al, 1995). PCNA expression in tumours is, however, known to be an unreliable measure of proliferative activity (Coltrera et al, 1993). In breast carcinoma, little correlation between p53 expression and tumour TLI was found (Silvestrini et al, 1994). Our relationship between high TLI and excess bcl-2 differs from Park et al (1995) who used flow cytometric proliferation data on non-small-cell lung cancers. Data relating p53 and bcl-2 to cell proliferation are scarce and inconclusive. More studies are needed to reach a meaningful conclusion.

High levels of, presumably non-functional, p53 should limit apoptosis and reduce tumour cell loss. Tumours with high p53 levels have more cell loss than cancers with low p53 levels. This was not, however, statistically significant in the small number of cases available for study. A similar trend (not statistically significant) with bcl-2 levels (high bcl-2 with high cell loss) was also against expectations. High bcl-2 levels, assuming maintainance of the protein's function, should suppress apoptosis and diminish cell loss.

In this study, most non-small-cell carcinomas tended to show negative or weak staining for bcl-2. In strongly positive tumours, staining was homogeneous. Pezzella et al (1993) documented bcl-2 positivity in 25% of squamous cell carcinomas and noted homogeneous staining but did not comment on staining intensity. Of interest was the strong bcl-2 staining in the three neuroendocrine tumours (two small-cell and one large-cell type), which were associated with high cell-loss factors, and one lesion of clear cell carcinoma. The overexpression of bcl-2 in small-cell carcinomas seems contradictory in a tumour characterized by prominent apoptosis and a high cell loss factor but frequent overexpression of bcl-2 in lung small-cell carcinoma has already been reported (Ben-Ezra et al, 1994; Jiang et al, 1995).

We found that excess bcl-2 expression correlated with strong p53 staining, primarily in tumours of neuroendocrine type. In our non-neuroendocrine group, mutually exclusive staining was observed in only two tumours (cases 1 and 10) but was relatively exclusive in three others (cases 8, 12 and 13). These results mirror the inverse relationship between p53 and bcl-2 expression found in breast (Leek et al, 1994; Silvestrini et al, 1994) and thyroid (Pilotti et al, 1994) carcinoma. This may be explained by the down-regulation of bcl-2 expression by p53 via a negative response element on the *bcl-2* gene (Miyashita et al, 1994*a*). However, at least in lung tumours of neuroendocrine origin, mutant p53 may not be able to down-regulate the *bcl-2* gene. These genes are known to function differently in different tumour types (Pietenpol et al, 1994).

Excess bcl-2 expression may promote the malignant phenotype by suppressing apoptosis (Lu et al, 1996). Mutation of p53 may have the same effect, facilitating unopposed cell proliferation. *Both* these abnormalities may have a compounding effect on the progression of neoplasia. This concurs with the known clinical behaviour of neuroendocrine, particularly small-cell, lung carcinoma. Such combined expressin may interfere with the control of cell death in a tumour (Miyashita et al, 1994*b*).

© Cancer Research Campaign 1997

Immunohistochemically demonstrated excess p53 protein may not necessarily reflect gene mutation (Hall and Lane, 1994). *bcl-2* gene mutation may also lead to a functionally inactive protein product, which may still be detectable by immunohistochemistry. The existence of Bax and related proteins further compounds the issue When overexpressed, Bax counteracts bcl-2 (Yin et al, 1994). It may be important to determine cellular levels of Bax. If Bax levels are low, even low levels of bcl-2 may be sufficient to rescue a cell from apoptosis. If, however, Bax is abundant a cell may be more vulnerable to a (p53-mediated?) signal to die and thus require high levels of functional bcl-2 to save it (Yin et al, 1994). Perhaps in lung neuroendocrine carcinoma, Bax levels are also very high.

This is a complex issue involving many inter-related factors, some of which cannot, as yet, be measured in material such as ours. It seems likely that cell death control may vary between tumours. This may have implications when chemoresponsiveness in small-cell lung cancer is considered.

### REFERENCES

Ben-Ezra JM, Kornstein MJ, Grimes MM and Krystal G (1994) Small cell carcinomas of the lung express the bcl-2 protein. *Am J Path*, **145**: 1036–1040

Collins VP, Leoffler RK and Tivey H (1956) Observation on growth rates of human tumours. *Am J Roentgenol* **76**: 988–1000

Coltrera MD, Skelly M and Gown AM (1993) Anti-PCNA antibody PC10 yields unreliable proliferation indexes in routinely processed, deparaffinized, formalin-fixed tissue. *Appl Immunohistochem* **1**: 193–200

Hall PA and Levison DA (1990) Review: assessment of cell proliferation in histological material. *J Clin Pathol* **43**: 184–192

Hall PA and Lane DP (1994) p53 in tumour pathology: can we trust immunohistochemistry? – revisited. *J Pathol* **172**: 1–4

Ikegaki N, Katsumata M, Minna J and Tsujimoto Y (1994) Expression of bcl-2 in small cell lung carcinoma cells. *Cancer Res* **54**: 6–8

Jiang S-X, Sato Y, Kuwao S and Kameya T (1995) Expression of bcl-2 protein is prevalent in small cell carcinomas. *J Pathol* **177**: 135–138

Kerr KM and Lamb D (1984) Actual growth rate and tumour cell proliferation in human pulmonary neoplasms. *Br J Cancer* **50**: 343–349

Kerr KM, Robertson AMG and Lamb D (1983) *In vitro* thymidine labelling of human pulmonary neoplasms. *Br J Cancer* **47**: 245–252

King G (1994) Microwave heating as a method of unmasking immunoglobulin light chain antigen in paraffin sections. *UK NEQAS Immunocytochemistry News* **3**: 11–12

Lane DP (1993) A death in the life of p53. *Nature* **362**: 786–787

Leek R, Kaklamanis L, Pezzella F, Gatter KC and Harris AL (1994) Bcl-2 in normal human breast and carcinoma association with oestrogen receptor positive, epidermal growth factor receptor-negative tumours and *in situ* cancers. *Br J Cancer* **69**: 135–139

Lu Q-L, Abel P, Foster CS and Lalani E (1996) bcl-2: role in epithelial differentiation and oncogenesis. *Hum Pathol* **27**: 102–110

Miyashita T, Harigai M, Hanada M and Reed JC (1994*a*). Identification of a p53-dependent negative response element in the bcl-2 gene. *Cancer Res* **54**: 3131–3135

Miyashita T, Krajewski S, Krajewska M, Wang H-G, Lin H-K, Hoffman B, Lieberman D and Reed JC (1994*b*) Tumour suppressor p53 is a regulator of bcl-2 and bax gene expression in vitro and in vivo. *Oncogene* **9**: 1799–1805

Park KO, Kim YC and Choi IS (1995) Relationship of bcl-2 gene expression and DNA ploidy, proliferative activity in non-small cell lung carcinoma. *Am J Resp Crit Care Med* **4**: A277

Pezzella F, Turley H, Huzu I, Tungekar MF, Dunnill MS, Pierce CB, Harris A, Gatter KC and Mason DY (1993) bcl-2 protein in non-small cell lung carcinoma. *N Engl J Med* **329**: 690–694

Piepentol JA and Vogelstein B (1993) No room at the p53 inn. *Nature* **365**: 17–18

Pietenpol JA, Papadopoulos N, Markowitz S, Willson JKV, Kinzler KW and Vogelstein B (1994) Paradoxical inhibition of solid tumour cell growth by bcl-2. *Cancer Res* **54**: 3714–3717

Pilotti S, Collini P, Rilke F, Cattoretti G, Del Bo R and Pierotti MA (1994) Bcl-2 protein expression in carcinomas originating from the follicular epithelium of the thyroid gland. *J Pathol* **172**: 337–342

Silvestrini R, Veneroni S, Daidone MG, Benini E, Boracchi P, Mezzetti M, Di Fronzo G, Rilke F and Veronesi U (1994) The bcl-2 protein: a prognostic indicator strongly related to p53 protein in lymph node-negative breast cancer patients *J Natl Cancer Inst* **86**: 499–504

Steel GG (1967) Cell loss as a factor in the growth rate of human tumours. *Eur J Cancer* **3**: 381–387

Wiethege TH, Voss B and Muller K-M (1995) Positive PCNA status in lung tumour specimens correlates with p53 tumour suppressor gene accumulation. *Am J Resp Crit Care Med* **4**: A278

Yang E and Korsmeyer SJ (1996) Molecular thanatopsis: a discourse on the bcl-2 family and cell death. *Blood* **88**: 386–401

Yin X-M, Oltvai ZN and Korsmeyer SJ (1994) BH1 and BH2 domains of Bcl-2 are required for inhibition of apoptosis and heterodimerisation with Bax. *Nature*, **369**: 321–323

© Cancer Research Campaign 1997